# A synergic approach for nutrient recovery and biodiesel production by the cultivation of microalga species in the fertilizer plant wastewater

**DOI:** 10.1038/s41598-019-55748-w

**Published:** 2019-12-13

**Authors:** Indu Ambat, Sabina Bec, Elina Peltomaa, Varsha Srivastava, Anne Ojala, Mika Sillanpää

**Affiliations:** 1 Department of Green Chemistry, School of Engineering Science, Lappeenranta University of Technology, Sammonkatu 12, FI-50130 Mikkeli, Finland; 2 Faculty of Biological and Environmental Sciences, Ecosystems and Environment Research Programme, University of Helsinki, Niemenkatu 73, FI -15140 Lahti, Finland; 3 Institute of Atmospheric and Earth System Research (INAR)/Forest Sciences, University of Helsinki, P.O. Box 27, FI-00014 Helsinki, Finland; 4 Helsinki Institute of Sustainability Science (HELSUS), Yliopistonkatu 3, 00014 Helsingin, yliopisto Finland

**Keywords:** Environmental biotechnology, Pollution remediation, Gas chromatography

## Abstract

The combination of wastewater treatment and biodiesel production using algal cultivation was studied in the present work. The two main goals of the work were achieved by the cultivation of freshwater microalgae such as *Chlamydomonas* sp., *Scenedesmus ecornis*, and *Scenedesmus communis* in two different dilutions of fertilizer plant wastewater (FWWD1 and FWWD2) collected from Yara Suomi Oy, Finland. The growth pattern of different algal species in FWWD1 and FWWD2 was observed. The effect of pH on biomass concentration, lipid content, biomass productivity, and lipid productivity by all three algal species in FWWD1 and FWWD2 were monitored. The maximum biomass concentration and productivity were observed in FWWD1 at pH7.5 for *Chlamydomonas* sp. and at pH 8.5 for *S. ecornis* and *S. communis*. The maximum lipid content was detected in *Chlamydomonas* sp at pH5.5, followed by *S. ecornis* and then *S. communis* at pH 7.5 in FWWD2 obtained after co-solvent extraction method. The most significant removal percentage of COD by all algal species were observed in FWWD1, whereas the highest removal percentage of TN and TP were detected in FWWD2, respectively. The fatty acid methyl ester (FAME) characterization of each algal species in FWWD1 and FWWD2 at their optimum pH was investigated to determine the quality of obtained biodiesel.

## Introduction

The scarcity of conventional fuels and pollution of water resources have been a global issue with the population growth^1,2^. Thus, it leads to the need for sustainable alternative fuels and reusing of wastewater using different treatment technologies^2,3^. Biodiesel composed of fatty acid methyl esters (FAME) serves as one of the viable alternative fuels. The animal fat, vegetable oil, and algal oil serves as sources for FAME production. The renewability, biodegradability, eco-friendly nature, and non-toxicity are the features possessed by FAME^4–7^.

In 1942, the concept of using algae for energy production was introduced by Harder and Witch. Later in 1955 Meier, and Ostwald and Golueke in 1960 used algal carbohydrates for production of methane (CH_4_) gas under anaerobic conditions. The energy production from algal species attained key impulsion in the early 1970s due to the increase in the cost of energy^6,8^. In current years, the microalgae serve as a potential source for the synthesis of biodiesel and also play a substantial role in the removal of nutrient from wastewater^3,6,9–14^. The algal species possess features such as high growth rate, high oil content, availability, the capability to reduce greenhouse gases (GHGs) in the atmosphere, and besides, algal production does not compete with food market which made them a favorable feedstock for biodiesel production^3,15–17^. In comparison with conventional methods such as chemical precipitation and biological nitrification/denitrification for wastewater treatment process algae offer efficient nutrient removal by the utilization of nutrients in wastewater for its growth and production of biomass for biodiesel production^2,6,12,13,18^. Therefore, biodiesel derived from algal biomass grown in wastewater medium serves as a promising sustainable, eco-friendly, and cost-efficient source of energy^2,12,14,16,19–21^.

In 2010, Flamos *et al*. stated that microalgae production has excellent potentials in economic dimensions as it can increase revenues and employment. The co-products and byproducts obtained as a result of biofuel production offer supplementary economic values. Furthermore, the income taxes of all countries can be raised because of microalgae production. Moreover, microalgae production can create openings in developing countries, and also biofuel based businesses can improve the energy security and dependency of different nations on crude oil imports^22^. Later in 2016, Hwang *et al*. reported that microalgae-based wastewater treatment systems demonstrate an eco-friendly and low-cost wastewater treatment alternative in comparison with conventional wastewater treatment processes^23^.

The biodiesel production from algal biomass includes different steps like algal cultivation, biomass collection, lipid extraction, and transesterification of lipids. The lipid extraction step is of extreme importance in the algal biodiesel production process. The most widely used methods for lipid extraction from algal species are solvent extraction, enzyme extraction, and mechanical extraction^2,16,21,24^. All these methods are energy demanding and also require large quantities of solvents. For commercialization and economical production of algal biodiesel it must be attained with low consumption of energy and solvents. Recently, ionic liquids (ILs) have got more considerable attention because of their thermal stability, non-volatile nature, low vapor pressure, shorter reaction time, specific stability, polarity, and high-performance yield. Hence, ILs act as a potential substitute for volatile solvents and also considered as greener solvents. ILs are salts sustained as a liquid at moderate to room temperature (0–140 °C) and they comprise of a nitrogen-containing ring structure (eg: pyrinidine or imidazolium) as cation to which a wide range of functional side groups can be attached^21,25,26^. In 2010 methanol was stated as right choice as polar covalent molecules (PCM) for lipid extraction by Young *et al*. and in 2014, 1-ethyl-3-methyl imidazolium diethyl phosphate, [Emim] DEP reported as a best ionic liquid by Choi *et al*.^21,25^.

The main objective of the present work is the simultaneous removal of nutrients and biodiesel production from wastewater collected from a fertilizer plant using *Chlamydomonas* sp., *Scenedesmus ecornis*, and *Scenedesmus communis*. To the best of our knowledge, the cultivation of these algal species in wastewater for biodiesel production has not been reported. Moreover, the lipid content of algal species used in this study is comparable to other algal species commonly used for biodiesel production and nutrient removal from various kinds of wastewater^27^. The earlier reported studies explored various kinds of wastewater, but fertilizer wastewater was rather scanty and not well explored^6^. Hence the growth of algal species in fertilizer wastewater (FWW) was examined. The nutrient removal efficiency of the algal species in the nitrogen-rich environment was investigated. Based on previously reported studies suggested that ILs and PCM combination showed better results for lipid extraction in a more eco-friendly way than consuming large volatile organic solvents^21,24–26^. Therefore, the extraction of lipids using a novel co-solvent system, [Emim] DEP ionic liquid coupled with PCM as methanol was performed. The influence of pH on biomass concentration, biomass productivity, lipid content, and lipid productivity of various algal species in fertilizer wastewater (FWW) was observed. The FAME composition and properties of the obtained biodiesel were studied.

## Materials and Methods

### Characterization of collected wastewater

The Fertilizer plant wastewater (FWW) was collected from Yara Suomi Oy, Siilinjärvi, Pohjois-Savo, Finland. The physicochemical properties of the collected wastewater sample were analyzed. The parameters of wastewater such as COD, total nitrogen (TN), and total phosphorus (TP) were analyzed according to standard methods (APHA, 2012) and measured with a spectrophotometer DR3900 (Hatch, Germany)^28^. The concentration of metals and the NH4-N, NO3-N in wastewater samples were analyzed using Agilent 5110 inductively coupled plasma (ICP) and using Hach Lange kits (Hach Lange, Germany) and DR 3900 spectrophotometer, respectively^1,29–31^.

### Pre-cultivation of algal species

The *S. communis, S. ecornis, and Chlamydomonas* sp. were given by one of the authors (E. Peltomaa, University of Helsinki, Finland). The Modified WC Medium (MWC) was used to pre-culture and maintain all the algal species. MWC contains the following ingredients: (1) chemicals such as CaCl_2_.2H_2_O (36.80 g L^−1^), MgSO_4_.7H_2_O (37.00 g L^−1^), NaHCO_3_ (12.60 g L^−1^), K_2_HPO_4_.3H_2_O (11.40 g L^−1^), NaNO_3_ (85.00 g L^−1^), Na_2_O_3_Si.5H_2_O (21.20 g L^−1^); (2) the combined trace elements EDTANa_2_ (4.36 g L^−1^), FeCl_3_.6H_2_O (3.15 g L^−1^), CuSO_4_.5H_2_O (0.01 g L^−1^), ZnSO_4_.7H_2_O (0.022 g L^−1^), CoCl_2_.6H_2_O (0.01 g L^−1^), MnCl_2_.4H_2_O (0.18 g L^−1^), Na_2_MoO_4_.2H_2_O(0.006 g L^−1^), H_3_BO_3_ (1 g L^−1^); (3) vitamin mix thiamine HCL (0.1 g L^−1^), biotin (0.0005 g L^−1^), cyanocobalamine (0.0005 g L^−1^); (4) TES buffer (0.115 g L^−1^). Each algal strain was grown in tissue culture flasks with MWC medium as triplicates in a growth cabinet (SANYO growth Chamber MLR-350 H; 294 L with white light source) at 20 °C with a continuous photon flux density (PFD) of 150 μmol m^−2^ s^−1^.

### Cultivation of algal species in wastewater samples

Initially, due to high nutrient concentrations the fertilizer plant wastewater samples were diluted 100 fold (FWWD1) and 200 fold (FWWD2), respectively. Carbon source (NaHCO_3_) in 250 mgL^−1^ concentration was introduced to each dilution of the wastewater sample for algal growth. The pH of the samples was adjusted to 7.5 before algal inoculation using sodium hydroxide. Later, the pH adjusted samples were autoclaved. The effect of pH on algal biomass production and lipid content were determined by conducting experiments at various pH such as 5.5, 7.5, and 8.5 correspondingly. The initial inoculum concentration was 0.20 gL^−1^, 0.21 gL^−1^, and 0.20gL^−1^ for *Chlamydomonas* sp., *S. communis, and S. ecornis*, respectively. All the three microalgae strains were cultivated as triplicates separately in batch culture flasks with a working volume of 500 ml for ten days at 25 °C with a continuous PFD of 150 μmol m^−2^ s^−1^.

### Analytical methods

#### Algal growth rate and biomass productivity

The optical density of each algal strain was observed on alternative days at 680 nm using the spectrophotometric method. The biomass of each strain was collected using centrifugation and followed by freeze-drying (Christ Alpha 2–4 LD plus). Later, the dry weight (DW) measurements of algal samples were performed to confirm the algal growth^9–11,14^. The biomass of each strain at the initial phase, log phase, and late log phase were determined gravimetrically and biomass productivity calculated as described in Eq. 1 ^10,12,14^.

1 $$Biomass\,productivity\,(mg{L}^{-1}{d}^{-1})=\frac{Biomass\,concentration\,(mg{L}^{-1})}{No.of\,days}$$

#### Nutrient removal efficiency of algal strains

The 30 ml of sample was collected from each culture separately on alternative days to examine the nutrient removal capability. The collected samples were centrifuged and filtered using 0.45 µm PTFE syringe filters from Van Waters and Rogers (VWR) international. The filtered samples were then investigated for TN, COD, and TP according to standard methods (APHA, 2012) and measured with a spectrophotometer DR3900 (Hatch, Germany)^28^. Nutrient removal efficiency of each algal strain was calculated using Eq. 2.

2 $$Removal\,percentage\,( \% )=\frac{Initial\,concentration-Final\,concentration}{Initial\,concentration}\times 100$$

#### Lipid extraction and lipid productivity

The lipid extraction of algal biomass was achieved with the help of the co-solvent system consisting of 1-ethyl-3-methyl imidazolium diethyl phosphate, [Emim] DEP and PCM (methanol). During the extraction process, 500 mg of dried algal biomass was dispersed in the co-solvent system prepared by mixing [Emim] DEP and methanol in 1.2:1 (v/v) ratio. The biomass-co-solvent system was mixed continuously for 18 h at 65 °C, and later, it was cooled at room temperature. The addition of hexane results in the extraction of lipids and centrifugation was conducted for the separation of ionic liquid- PCM phase and lipid-containing hexane phase. The hexane was removed by rotary evaporator for lipid recovery^21,25,26^. Later obtained lipids were freeze-dried, and the total lipids in each algal strain obtained using the co-solvent system were determined gravimetrically, and the lipid content of each algal strain was expressed as a percentage of DW, respectively. The lipid productivity of each strain was calculated using Eq. 3 ^10,14,26^.

3 $$Lipid\,productivity\,(mg{L}^{-1}{d}^{-1})=Biomass\,productivity\times \frac{Lipid\,content}{100}$$

#### FAME characterization

Typically, not all kinds of lipids in algae cannot be converted to fatty acid methyl esters (FAME), for example, glycolipids and phospholipids. Hence, the conversion of algal lipids to FAME and characterization of the obtained FAME is relevant to measure the content of lipids that are convertible to FAMEs^10,30^. The base-catalyzed transesterification of lipids was performed using 2 M potassium hydroxide (KOH) in analytical grade methanol. The supernatant was collected when the phase separation of the samples was attained. The obtained supernatant was subjected to gas chromatography with mass spectrometry (GC-MS) analysis. During the investigation of ester, FAME mix C4–C24 and pentadecanoic acid methyl ester (Sigma-Aldrich) were used as a quantitative standard and as an internal standard respectively^32–34^. The obtained FAME was analyzed by Agilent GC-MS (GC6890N, MS 5975) with DB-wax FAME column (30 m, 0.25 mm, 0.25 µm). The inlet temperature was 250 °C in split mode. The oven temperature was programmed at 50 °C for 1 min, and it raised at the rate of 25 °C/min to 200 °C and 3 °C /min to 230 °C and it was held there for 23 min. The FAME composition was recognized and quantified using the National Institute of Standards and Technology (NIST) 2014 MS library and GCMS chromatogram^29,35^. The properties of algal FAME such as Iodine value (IV), saponification value (SV), and cetane number (CN) were determined using empirical formulas given below^36–39^.

4 $$Cetane\,number\,(CN)=46.3+\frac{5458}{SV}-0.225\times IV$$

Where saponification value (SV) are denoted in mg KOH g^−1^ and iodine value (IV) is presented in g I 100 g^−1^.

The Eqs. 5 and 6, represent the empirical formula for the calculation of iodine value and saponification value of algal FAME respectively^37–39^.

5 $$IV=\varSigma \frac{254\times F\times D}{MW}$$

6 $$SV=\varSigma \frac{560\times F\times D}{MW}$$

where, F is the percentage weight of each fatty acid, D is the total number of double bonds and MW is the molecular weight of the respective fatty acid.

All the experiments were performed in triplicates and average values were reported. The obtained triplicate results were performed with EXCEL (Microsoft Office Enterprise, 2016) and analysis of variance (ANOVA) for presented data was determined using MATLAB R2017a version.

## Results and Discussion

### Wastewater characterization

The characteristic features of fertilizer plant wastewater (FWW) are shown in Table 1. The total phosphorus (TP), chemical oxygen demand (COD), total nitrogen (TN), and pH of wastewater samples were monitored before algal cultivation. The collected wastewater had 8200 mgL^−1^ of TN and 200 mgL^−1^ TP and pH of 5.5. The COD level of undiluted wastewater sample was 250 mgL^−1^.

**Table 1:** The characterization of fertilizer plant wastewater.

Parameters	Units	Concentration
pH	—	5.5
COD	mgL^−1^	250
TN	mgL^−1^	8200
NH_4_-N	mgL^−1^	4300
NO_3_-N	mgL^−1^	3900
TP	mgL^−1^	200
K	mgL^−1^	500

### Algal growth and biomass production

**Figure 1:**
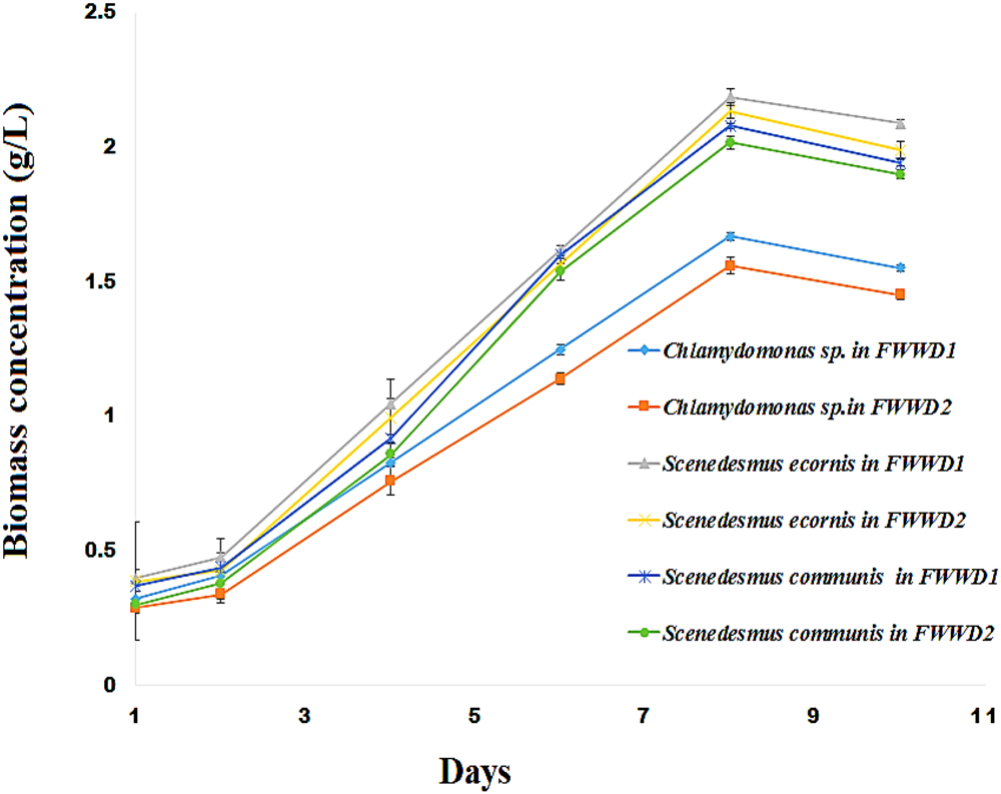
Growth curves of *Chlamydomonas* sp., *S. communis, and S. erconis in FWWD1 and FWWD2 of pH 7.5* at 25 °C with a continuous PFD of 150 μmol m^−2^ s^−1^ (mean ± SD).

The growth behavior of *Chlamydomonas* sp., *S. ecornis*, and *S. communis* in FWWD1, and FWWD2 were described in (Fig. 1). Each curve represents the growth phase of each algal species in wastewater samples. The algal species showed a lag phase of two days in fertilizer plant wastewater. The algal species showed higher biomass concentration in FWWD1 compared FWDD2 due to the high amount of nutrients^14,40^. Subsequently, the algal growth entered to an exponential phase after day two where, *Chlamydomonas* sp*., S. communis, and S. ecornis* exhibited a significant rise in biomass production up to day eight. The *Chlamydomonas* sp., *S. ecornis*, and *S. communis* showed maximum biomass concentration of 1.66 ± 0.028 gL^−1^, 2.19 ± 0.012 gL^−1^, 2.08 ± 0.017 gL^−1^ in FWWD1 whereas 1.56 ± 0.014 gL^−1^, 2.14 ± 0.032 gL^−1^, 2.02 ± 0.011 gL^−1^ in FWWD2 respectively. The growth behavior of each alga might be due to the effect of COD, nitrogen, and phosphorus concentration on each algal species^6,14,40,41^. The nutrients such as N, P, and K contribute major percentage of algal biomass^42,43^ and it was higher in FWWD1 (100 fold diluted wastewater) than in FWWD2 (200 fold diluted wastewater). Moreover, the lower carbon source (NaHCO_3_) to TN ratio due to high amount of TN in FWWD1 and high NPK amount in FWWD1 favored biomass production. The ANOVA analysis of variance of biomass concentration results showed a p-value lower than 0.05 is enough to conclude that biomass concentrations are significantly different for dilutions such as FWWD1, FWWD2 for different algal species.

**Figure 2:**
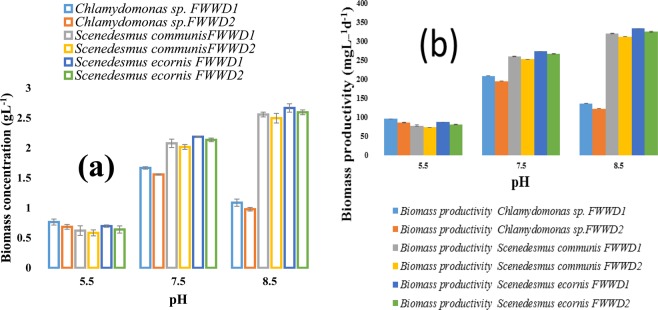
Effect of pH on (**a**) algal biomass production, and (**b**) biomass productivity *in FWWD1 and FWWD2* at 25 °C with a continuous PFD of 150 μmol m^−2^ s^−1^ (mean ± SD).

The effect of pH on algal biomass production, biomass productivity in FWWD1, and FWWD2 at 25 °C with a continuous PFD of 150 μmolm^−2^s^−1^ is depicted in Fig. 2. The biomass concentration is depended on the pH of the culture medium because it influences the efficiency of nutrient absorption and photosynthesis of the algal species. Besides, the pH range 6–8 is suitable for algal cultivation, whereas low pH causes enzyme inhibition in photosynthesis process^44–47^. *Chlamydomonas* sp. showed maximum biomass concentration and biomass productivity at pH 7.5 whereas *S. ecornis*, and *S. communis* had maxima at pH 8.5 in FWWD1, and FWWD2 illustrated in Fig. 2a,b ^44–46^. All algal species showed maximum biomass concentration and productivity in FWWD1 samples. The biomass productivity of 209 ± 2.68 mgL^−1^d^−1^ and 195 ± 2.29 mgL^−1^d^−1^ were displayed by *Chlamydomonas* sp. in FWWD1, and FWWD2 correspondingly. *S. ecornis* showed a maximum biomass concentration and productivity of 2.70 ± 0.071 mgL^−1^ and 334 ± 1.84 mgL^−1^d^−1^ in FWWD1 while *S. communis* exhibited 2.60 ± 0.044 mgL^−1^ and 320 ± 1.25 mgL^−1^d^−1^ of biomass concentration and productivity in FWWD1 respectively. *S. ecornis and S. communis* showed biomass productivity of 313 ± 2.0 mgL^−1^d^−1^ and 325 ± 1.42 mgL^−1^d^−1^ in FWWD2 correspondingly. The results also indicated that there were significant (P < 0.05) differences between the dilutions (FWWD1 and FWWD2) and pH used for cultivation for different algal species.

### Lipid content and productivity

**Figure 3:**
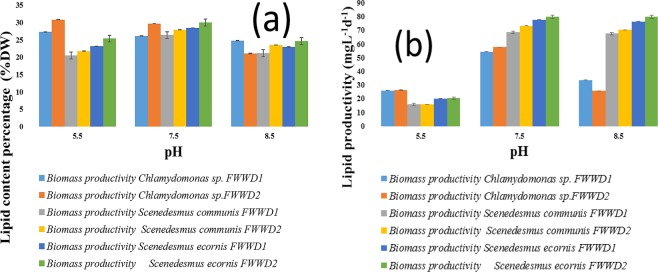
(**a**,**b**) Lipid content and productivity of different algal strains after co-solvent extraction (mean ± SD).

The maximum lipid content of 30.9 ± 0.085% was observed in *Chlamydomonas* sp. at pH 5.5 followed by *S. ecornis* (30.0 ± 0.062%) and *S. communis* (27.9 ± 0.034%) at pH 7.5 in FWWD2 (Fig. 3a). All algal strains exhibited maximum lipid content and productivity in FWWD2. Generally, under stressed conditions such as pH, nutrient (nitrogen or phosphorus), starvation leads to the accumulation of lipids in microalgae. Under normal growth conditions, the microalgae use the ATP and NADPH are produced during photosynthesis for biomass concentration. As a result, ADP and NADP^+^ are accessible as the electron acceptor for photosynthesis. During nutrients limited conditions lead to depletion of NADP^+^ due to reduced cell growth and proliferation. The photosynthesis process is regulated by the abundance of light; therefore, it cannot be switch off completely. At this stage, fatty acid biosynthesis or triglycerides production act as a cell protective mechanism NADP^+^ by the consumption NADPH^48–50^. The channelization of carbohydrates from the growth phase to TGA production was also possible during limited nutrient conditions^51^. The TGA production and accumulation can be enhanced by alteration in pH, and it due to the adaptive mechanism of algae towards the pH of culture medium. Based on previously reported studies, *Chlamydomonas* sp. showed maximum lipid production under acidic conditions, whereas *Scenedesmus* sp. under slightly alkaline conditions due to their morphological difference^45,46,48^. The maximum content of lipids in algal species grown in FWWD2 is possibly due to the lower amount of nutrients and higher carbon source (NaHCO_3_) to TN ratio compared to FWWD1 depicted in Fig. 3a ^6,41,52,53^. Moreover, the smaller amount of nitrogen in FWWD2 in comparison to FWWD1 could have enhanced the lipid content^40,48,53^. The maximum lipid productivity by all algal strains was observed at pH7.5. *Chlamydomonas* sp.*, S. ecornis*, and *S. communis* showed maximum lipid productivity of 57.8 ± 1.87 mgL^−1^d^−1^, 80 ± 2.04 mgL^−1^d^−1^, and 73.5 ± 1.66 mgL^−1^d^−1^, respectively illustrated in Fig. 3b. The p-values obtained after ANOVA analysis of variance are smaller than 0.05 for mean results of lipid content and lipid productivity shows that obtained results were indicated significant differences in different dilutions (FWWD1 and FWWD2) for different algal species.

### Nutrient removal

The previous studies show that treatment of various kinds of wastewater was possible using algal species^6,52,54^. The COD, N, and P removal mainly observed due to the consumption of microalgal cells during the growth process. Generally, algal cells adsorb inorganic nitrogen in the form of nitrate after the nitrification process or continue to be degraded into gas nitrogen^55,56^. The TP removal can be achieved by adsorption, ion exchange, or precipitation. The microalgae can degrade phosphate to some extent via microbial activities and besides microalgae, the accumulation of precipitated phosphorus during algal cultivation with a rise in pH^57,58^.

**Figure 4:**
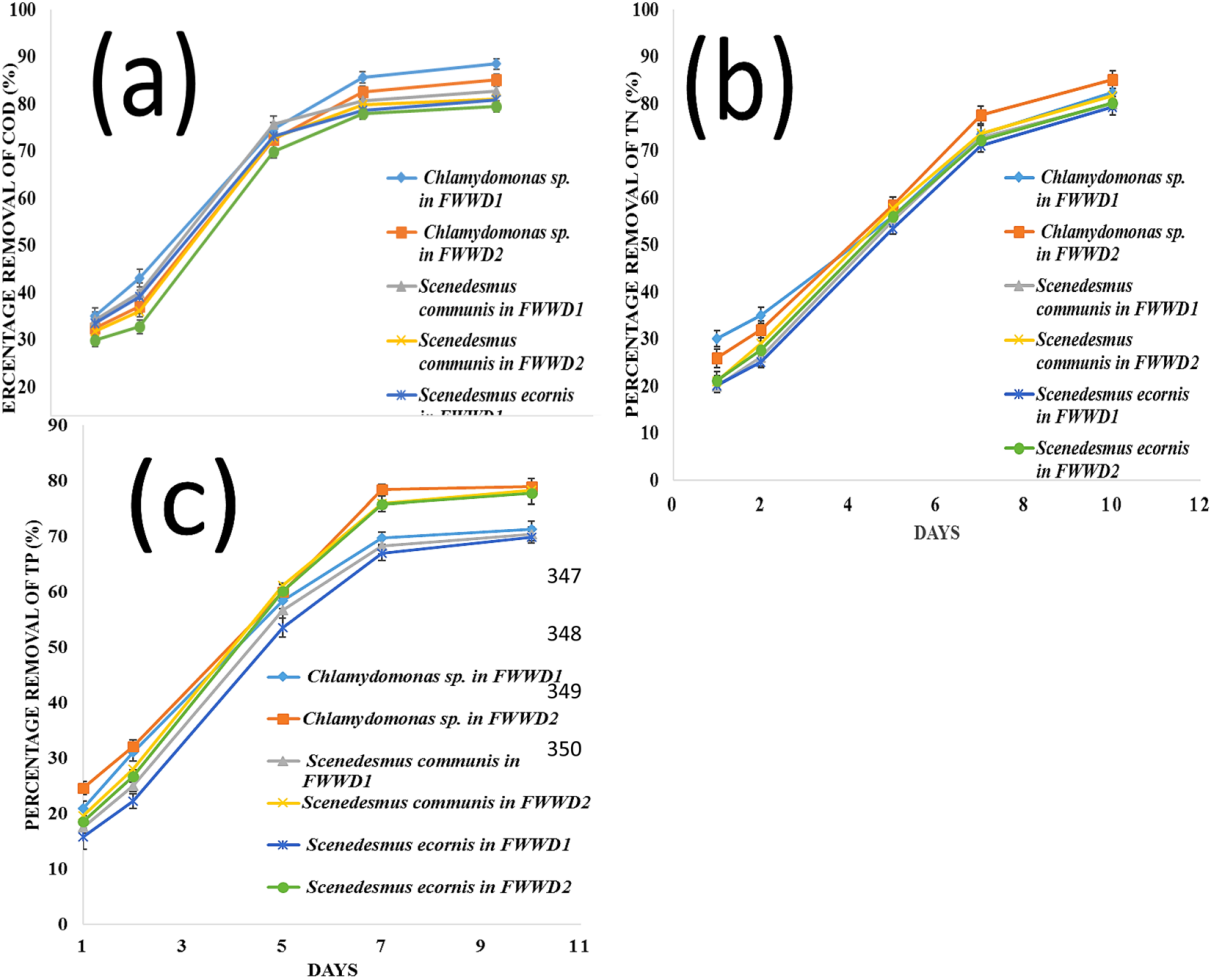
The fertilizer plant wastewater treatment performance by *Chlamydomonas* sp., *S. ecornis*, and *S. communis* at 25 °C with a continuous PFD of 150 μmol m^−2^ s^−1^ at pH 7.5 as percentage removal of (**a**) COD (**b**) TN and (**c**) TP (mean ± SD).

The current work indicates a drastic removal of COD in early days of algal cultivation (Fig. 4a). *Chlamydomonas* sp., *S. communis*, and *S. ecornis* exhibited higher removal of COD in FWWD1. The more significant removal of COD in FWWD1 than in FWWD2 is possibly due to the lower carbon source (NaHCO_3_) to TN ratio due to high amount of TN in FWWD1^41,59^. The maximum percentage of COD removal by *Chlamydomonas* sp, *S. communis*, and *S. ecornis* were 88.6 ± 1.1%, 82.7 ± 1.4%, and 80.9 ± 1.6% respectively within ten days (Fig. 4a).

The maximum percentage removal of TN in FWWD2 by *Chlamydomonas* sp, *S. communis*, and *S. ecornis* were 85.2 ± 1.6%, 81.8 ± 1.3%, and 80.2 ± 2.04% correspondingly (Fig. 4b). *Chlamydomonas* sp, *S. communis*, and *S. ecornis* showed 82.5 ± 2.02%, 80 ± 1.7%, and 79.3 ± 1.3% removal of TN in FWWD1, respectively within ten days represented in Fig. 4b. All the algal strains showed maximum removal of TN in FWWD2. The maximum percentage removal of TN in FWWD2 is possibly due to the lower concentration of nitrogen in FWWD2 compared FWWD1^41,59,60^.

Within ten days of cultivation, *Chlamydomonas sp*, *S. communis*, and *S. ecornis* in FWWD2 showed 79 ± 1.3%, 78.3 ± 1.1%, and 77.8 ± 1.6% removal of TP respectively (Fig. 4c). The TP removal was 71.4 ± 1.4%, 70.5 ± 1.9%, and 69.9 ± 1.2% by *Chlamydomonas* sp, *S. communis*, and *S. ecornis* in FWWD1 correspondingly shown in Fig. 4c. The TP removal was higher in wastewater samples with a low concentration of nutrients^10,60^. The smaller amount of nutrients might be the reason for increased removal percentage of TP in FWWD2 related to FWWD1. The COD, TN and TP removals within 10 days were significantly (P < 0.05) different among different algal species for different dilutions (FWWD1 and FWWD2).

### Composition and properties of FAME

The FAME profile of *Chlamydomonas* sp*., S. ecornis*, and *S. communis* mainly consisted of saturated fatty acid methyl ester such as palmitic acid methyl ester (C16:0), stearic acid methyl ester (C18:0) and unsaturated fatty acid methyl ester like palmitoleic acid methyl ester (C16:1), oleic acid methyl ester (C18:1n9c), linoleic acid methyl ester (C18:2n6c), and α –linolenic acid methyl ester (C18:3n3) (Table 2). The FAME yields 16%, 10.5%, and 8.9% of DW were correspondingly attained by *Chlamydomonas* sp*., S. ecornis* and *S. communis* grown in FWWD2 at their optimum pH. The proportion of saturated fatty acid (% DW) was higher in fertilizer plant wastewater than in MWC medium. The increased saturated fatty acid content in *Chlamydomonas* sp. was observed in weakly acidic conditions whereas for *Scenedesmus* sp more significant amount of saturated fatty acid in slightly alkaline conditions of the wastewater samples^53,61^. Furthermore, the algae showed the rise in the amount of triglycerides (TGA), the increase in the concentration of oleic fatty acid and reduction in linolenic fatty acid amount in FWWD2, which was due to the lower level of nitrogen and phosphorus in the medium^34,53^.

**Table 2:** Of FAME composition and properties of different algae in MWC, FWWD1, and FWWD2 within ten days.

FAME Composition	Chlamydomonas sp.			S. ecornis			S. communis		
	MWC	FWWD1	FWWD2	MWC	FWWD1	FWWD2	MWC	FWWD1	FWWD2
**Saturated fatty acids (% of total FAME)**									
C16:0	24.37	30.51	33.44	33.05	37.45	38.56	20.4	23.12	25.51
C18:0	5.16	4.4	3.2	4.1	4.5	3.5	3.29	4.50	3.40
**Unsaturated fatty acids (% of total FAME)**									
C 16:1	1.35	1.29	2.59	1.05	1.55	1.79	1.28	1.15	2.59
C18:1n9c	8.75	9.27	22.60	34.98	33.59	38.24	21.09	19.88	23.69
C18:2n6c	20.86	19.30	18.98	2.36	3.44	2.51	6.55	5.16	5.57
C18:3n3	16.17	17.24	6.43	14.55	12.09	10.67	15.75	14.2	13.95
Total FAME (%DW)	8.89	10.05	15.99	8.83	9.88	10.46	6.6	7.07	8.95
Iodine value (g I 100 g^−1^)	91.24	91.75	74.87	76.58	71.06	69.91	75.20	67.22	72.14
Cetane number	60.15	57.81	59.66	58.43	58.76	58.23	68.25	70.12	65.45

The lower amount of unsaturated fatty acid or drop in linolenic fatty acid in algal species grown in fertilizer wastewater plant increases oxidation stability^3,53,62^. The lesser amount of long chain fatty acid reduces filter clogging at the lower temperature^3,63,64^. The increase in unsaturated fatty acid content reduces the pour point of fuel (Zhu *et al*., 2013). The calculated iodine value and cetane number of all algal FAME were within EN 14214 limits^3,38^.

## Conclusions

The treatment of fertilizer plant wastewater was excellently performed by *Chlamydomonas* sp., *S. ecornis*, and *S. communis*. The biomass concentration, lipid content, and FAME composition depends on the pH and level of nutrients. In comparison with Chlamydomonas sp., the biomass concentration of *S. communis* and *S. erconis* were higher at alkaline pH. Moreover, the biomass concentration and productivity of algae were maximum in wastewater samples with an increased level of nutrients. *Chlamydomonas* sp. showed the most significant lipid production at pH5.5 whereas *S. communis* and *S. erconis* at pH 7.5. *Chlamydomonas* sp., *S. communis*, and *S. ecornis* showed maximum removal of COD in wastewater samples with the highest concentration of nutrients while TN and TP removal were most significant in wastewater samples with the lower level of nutrients. All algaeshowed maximum total FAME (%DW) in wastewater samples with the smallest amount of nutrients. The entire FAME (%DW) was highest in the following order (a) *Chlamydomonas* sp., (b) *S. erconis*, and (c) *S. communis*. The fuel properties of the obtained algal FAME are suitable for proper fuel. The results of the current work showed a collaborative growth of microalgae in fertilizer wastewater and cost-effective production biodiesel from the obtained algal biomass. Thereby this approach offers a sustainable solution for energy production, wastewater treatment, and nutrient recovery.
